# Berberine suppresses colon inflammation via integrated modulation of host metabolism, microbial ecology, and innate immune signaling

**DOI:** 10.7150/thno.116546

**Published:** 2026-01-01

**Authors:** Yaqin Xiao, Xueying Li, Yuanyuan Fang, Miao Guo, Mingju Shui, Guofeng Zhong, Hefeng Zhou, Chengyuan Lin, Baofa Sun, Shengpeng Wang

**Affiliations:** 1 State Key Laboratory of Mechanism and Quality of Chinese Medicine, Institute of Chinese Medical Sciences, University of Macau, Taipa, Macao, China.; 2 State Key Laboratory of Medicinal Chemical Biology, Frontiers Science Center for Cell Responses, College of Life Sciences, Nankai University, Tianjin, China.; 3 Department of Bioengineering, Zhuhai Campus of Zunyi Medical University, Zhuhai, China.; 4 Centre for Chinese Herbal Medicine Drug Development, Hong Kong Baptist University, Hong Kong, China.

**Keywords:** Berberine, inflammation, single-cell, *Akkermansia*, Interleukin-1β

## Abstract

**Background:** Berberine, a natural compound with unique bioactivity, has been widely used in the treatment of gastrointestinal inflammatory diseases. Despite its well-documented anti-inflammatory properties, the system-level regulatory network underlying its multifaceted mechanisms remains poorly understood.

**Methods:** In this study, we employed a multi-level analytical approach, integrating single-cell RNA sequencing, targeted metabolomics, 16S rRNA gene sequencing, and drug-target analysis, to elucidate the integrative effects of berberine on gut microbiota-metabolism-immune interactions.

**Results:** Single-cell RNA sequencing revealed that berberine enhances energy metabolism in intestinal cells of DSS-induced mice, thereby maintaining normal physiological functions. Targeted metabolomics analysis of short-chain fatty acids, combined with 16S rRNA gene sequencing, demonstrated that berberine supplementation significantly increases short-chain fatty acid (SCFA) levels in the intestinal environment and selectively enriches the abundance of *Akkermansia*. Furthermore, single-cell RNA sequencing data indicated that berberine inhibits fibroblast-to-lymphatic transformation and suppresses the expression of interleukin-1*β*, leading to reduced immune activation in innate immune cells. Drug-target analysis identified shared molecular targets between berberine and various immunotherapeutic agents.

**Conclusion:** This study provides a comprehensive understanding of berberine's multi-target mechanisms and highlights its potential as a therapeutic agent for inflammatory diseases through the modulation of gut microbiota, host metabolism, and immune responses.

## Introduction

Berberine, a benzyl tetrahydroisoquinoline alkaloid (C₂₀H₁₈NO₄), is naturally occurring in the roots and rhizomes of Coptis chinensis and related medicinal plants 1. Berberine has multiple pharmacological effects, including antioxidant 2, antibacterial 3, and anti-inflammatory properties 4. Mechanistic studies have established its capacity to modulate key inflammatory pathways including NF-κB and JAK/STAT signaling 5,6, underpinning its therapeutic potential for gastrointestinal disorders. Clinical evidence supports the application of berberine in managing gastric ulcers, gastritis, and enteritis 7,8, attributed to its favorable safety profile and cost-effectiveness. Recent investigations further highlight the emerging role of berberine in intestinal disease prevention and treatment 9, warranting deeper exploration of its cellular mechanisms.

Ulcerative colitis (UC) imposes substantial burdens through chronic gastrointestinal inflammation and progressive tissue damage, often culminating in irreversible complications. Affected individuals may develop pancolonic involvement or persistent colonic remodeling, resulting in functional impairment and diminished quality of life 10. The pathophysiology of UC originates from a triad of interrelated processes: compromised intestinal barrier integrity disrupts mucosal homeostasis, facilitating pathogenic invasion. This breach triggers neutrophil infiltration and extracellular trap deposition at epithelial injury sites 11, which exacerbate immune hyperactivity via overproduction of pro-inflammatory mediators including TNF-*α* and IL-1*β* 12,13. Concurrent microbial dysregulation manifests as reduced populations of short-chain fatty acids (SCFAs) producing *Bacteroidetes* and *Firmicutes* 14, thereby depriving colonocytes of their primary metabolic substrates. The synergistic interplay between barrier dysfunction, immune activation, and microbial imbalance establishes a pathogenic loop that sustains chronic inflammation. The dextran sulfate sodium (DSS) induced murine model of UC has been widely adopted to facilitate the study of this disease process. First, it recapitulates many key features of human UC, including diarrhea, bloody stools, weight loss, and mucosal ulceration 15. Second, by adjusting the dosage regimen of DSS, this model enables the induction of not only the acute phase but also the chronic phase of UC 16. Owing to its reproducible time course, consistent disease development and severity among animals, and relatively uniform lesion distribution, the DSS induced murine model represents a valuable and reliable experimental system.

To delineate the anti-inflammatory mechanism of berberine, we implemented a longitudinal multi-omics strategy in DSS-induced mice. Integration of single-cell transcriptomics, gut microbiota profiling, and SCFAs metabolomic revealed dual therapeutic actions: (i) microbial metabolic reprogramming enhancing colonocyte energy supply, and (ii) direct suppression of innate immune signaling pathways. Physiological assessments confirmed the functional relevance of these molecular changes, demonstrating improved barrier integrity and attenuated inflammatory responses. Furthermore, the drug-target analysis expanded the potential applications of berberine.

## Materials and Methods

### Animal experiment designs

Specific pathogen-free male C57BL/6J mice (6-week-old) were purchased from Guangdong Zhiyuan Biotechnology Company Limited, and maintained with a temperature at 22 °C and with a 12-h dark-light cycle. All animal experiments were conducted in strict accordance with protocols approved by the Animal Experiment Ethics Committee of Zunyi Medical University. Mice were acclimated for one week, with free access to a regular chow diet and sterile drinking water before the start of the formal experiment. After acclimation, the DSS, 5-ASA, and berberine groups (n = 5 per group) received 2.5% DSS in their drinking water for 7 days, while being orally administered 0.2 mL of water, 60 mg/kg 5-ASA, and 60 mg/kg berberine, respectively. The Con group (n = 5) received normal water and was given 0.2 mL of water daily. The severity of colitis was assessed by monitoring the body weight, diarrhea, and hematochezia of mice daily and recorded using the disease activity index (DAI) scoring system 17.

### Assessment of intestinal barrier integrity *in vivo*

On the 8^th^ day, after 6 h of fasting, mice were gavaged with 0.6 g/kg 4-kDa fluorescein isothiocyanate-dextran (FITC-dextran, Sigma). After 4 h, the mice were anesthetized with 70 mg/kg pentobarbital sodium via intraperitoneal injection, followed by blood collection through cardiac puncture. The fluorescence intensity of FITC in the serum was measured with excitation/emission wavelengths of 485 nm and 520 nm, respectively.

### Tissue histology and immunostaining

Colon tissue samples were fixed in 4% paraformaldehyde (Beyotime), followed by paraffin embedding, sectioning, and hematoxylin-eosin (H&E) staining. Histological damage to the colon was evaluated according to the Geboes score system 18.

Deparaffinized colon sections were blocked with 5% bovine serum albumin (Beyotime) for immunofluorescence analysis for 30 min. Then, the specific primary antibody was added and incubated overnight at 4 °C. Antibodies used: anti-IL-1*β* (1: 100, Servicebio), anti-F4/80 (1: 100, Servicebio), anti-occludin (1: 500, Servicebio), anti-ZO-1 (1: 500, Servicebio) and anti-Muc2 (1: 100, Servicebio). Subsequently, the slides were incubated with a CY3-conjugated goat anti-rabbit IgG secondary antibody (Servicebio, 1: 300) at room temperature for 50 min in the dark. Nuclei were stained with 4′,6-diamidino-2-phenylindole (DAPI, Beyotime) for 10 min. All analyses were performed using Caseviewer software.

### Cytokine profiling by enzyme-linked immunosorbent assay (ELISA)

Quantification of cytokines in colon tissue was conducted using ELISA kits for IL-1*β* (Biolegend) and TNF-*α* (Biolegend), along with Pierce BCA protein assay kits (Thermo), following the manufacturer's protocols.

### Targeted quantification of SCFAs by GC-MS metabolomics

Mouse feces (20 mg) were mixed with 800 μL of a 0.5% phosphoric acid solution containing 10 μg/mL of 2-ethylbutyric acid. The resultant suspension was centrifuged (10,000×g, 5 min, 4 °C) and filtered through 0.22 μm nylon membranes prior to derivatization.

SCFA analysis was performed using an Agilent 8890B-7000D GC/MSD system fitted with an HP-FFAP capillary column. The carrier gas was high-purity helium, set to a flow rate of 1.0 mL/min. The instrument parameters were configured as follows: injection port: 180 °C (1 μL, split 10:1); oven program: 80 °C to 120 °C at 20 °C/min, then to 160 °C at 5 °C/min, hold at 220 °C for 3 min; Detection: selected ion monitoring (SIM). Data processing was performed with Masshunter quantitative software (v.10.0.707.0) for automated SCFA ion integration.

### 16S ribosomal RNA gene sequencing and data analyses

Mouse feces (100 mg) were used to extract DNA according to the OMG-Soil DNA Kit (Omega Bio-Tek Georgi) instructions. DNA was amplified by PCR targeting the variable regions 3 and 4 (V3-V4) of the 16S rRNA gene using the primer pairs 338F (5'-ACTCCTACGGGAGGCAGCAG-3') and 806R (5'-GGACTACHVGGGTWTCTAAT-3'). For sequence analysis, sequencing libraries were constructed with the NEXTflex Rapid DNA-Seq Kit (Bioo Scientific) and subjected to sequencing on an Illumina NovaSeq PE250 platform. Raw reads underwent quality control filtering with fastp (v.0.20.0) and were merged using FLASH (v.1.2.7). Chimeric sequences were removed prior to clustering operational taxonomic units (OTUs) at a 97% similarity threshold with the UPARSE (v.7.1) 19. The taxonomy was conducted for each OTU representative sequence using RDP classifier (v.2.2), aligning against the Silva 16S rRNA database (v.138) with a 70% confidence threshold. The valid sequence number list of the samples can be found in Table S1.

The similarity between microbial communities in various samples was analyzed through principal coordinate analysis (PCoA) using Bray-Curtis dissimilarity, performed with the Vegan v.2.5-3 package. *α*-diversity indices, including the Chao (richness) and Shannon (diversity) indices, were calculated with Mothur software (v.1.30.1) 20. Taxonomic classification was performed by aligning non-redundant gene set amino acid sequences against the NR database using BLASTP in Diamond 21.

### Single-cell library preparation and sequencing

Fresh mouse colon tissues were dissected into 2-3 mm^3^ fragments. Following the 10x Genomics Single Cell protocol, cell suspension with a concentration of around 1000 cells/μL and an overall cell viability exceeding 85% was prepared (Trypan Blue exclusion). Single-cell suspensions were loaded onto a Chromium microfluidic chip (10x Genomics) using the v3 chemistry kit for cell barcoding following encapsulation on a 10x Chromium Controller. Reverse transcription was then performed to convert the barcoded mRNA into cDNA. The cDNA was purified and subsequently sequenced using the Illumina NovaSeq 6000 platform (Illumina, USA).

### scRNA-seq quality control and data processing

Raw sequencing reads produced by the Illumina pipeline in FASTQ format were aligned to refdata-gex-mm10-2020-A and quantified as unique molecular identifiers (UMIs) using Cell Ranger count. The Cell Ranger output was loaded into Seurat (v.3.1.1) 22 with the following quality control criteria: (1) cells must express at least 200 gene features, and each gene feature must be present in at least three cells; (2) doublets and triplets identified by DoubletFinder were removed, and (3) low-quality cells with mitochondrial content exceeding 20% were filtered. The processed data were subjected to dimensionality reduction and unsupervised clustering for downstream analysis.

### scRNA-seq analysis

The top 3,000 variable genes were used in principal component analysis; 1-30 principal components were used in the FindNeighbors function. The FindClusters function was used to identify clusters. According to the reference 23,24, cluster names were determined by manually inspecting the lists of cluster marker genes. For the sub-clustering of cells, we followed a similar procedure. Marker genes and differentially expressed genes (DEGs) for each cluster were identified using the FindMarkers function, and only genes with adjusted *P* values less than 0.05 and log_2_(fold change) greater than 0.25 were considered. clusterProfiler (v.4.1.32) 25 software was used for Gene Ontology analysis, and GSEA (v.4.1.0) software with the MSigDB (v.7.5.1) were used to identify a priori-defined gene sets that show statistically significant differences between two groups. Pathways with *P* values less than 0.05 were considered as significantly enriched. Pathway scoring was based on the AUCell software (v.4.1.0). CellChat software (v.2.1.7) 26 was used to identify specific ligand-receptor pairs between two given clusters based on the scRNA-seq data. The R package monocle 27 was used to reconstruct single-cell pseudotime trajectories in fibroblasts, with genes exhibiting a false discovery rate (FDR) of less than 5% considered significantly varying with pseudotime. DisGeNET (v.7.0) 28 from Cytoscape (v.3.9.1) was utilized to identify disease-gene association. Drug-gene interactions were analyzed using the Drug-Gene Interaction Database 29 (DGIdb v.4.2.0), which mines the druggable genome across multiple drug databases, selecting only FDA-approved drugs.

### Statistical analysis

GraphPad Prism 6.0 statistical software was employed for statistical analysis. Error bars represent the standard deviation of the means in all Figures and statistical significance was assessed by one-way ANOVA followed by Tukey's multiple comparison test, with a two-sided *P* value < 0.05 considered significant.

## Results

### Berberine restricts colitis in DSS-induced mice

**Figure 1:**
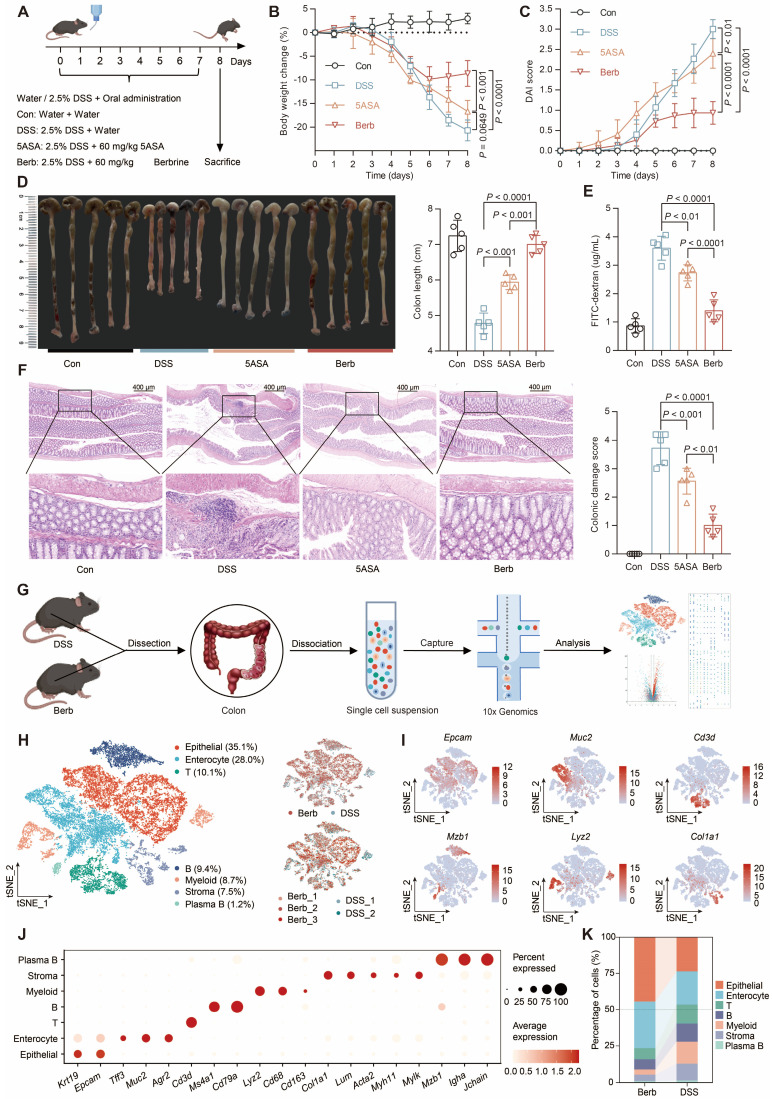
Berberine ameliorates DSS-induced colitis and modulates colonic cellular heterogeneity. (A) Experimental design schematic: C57BL/6J mice were provided with either water or water containing 2.5% DSS for 7 days and were orally administered either water, 50 mg/kg of 5-ASA, or berberine (*n* = 5 per group). (B) Longitudinal body weight changes expressed as percentage baseline. (C) Disease activity index (DAI) scoring integrating weight loss, stool consistency, and rectal bleeding. (D) Postmortem colon length quantification. (E) Serum FITC-dextran levels reflecting intestinal permeability. (F) Histopathological scoring of H&E-stained colon sections. Data represent mean ± SD (n = 5 biological replicates). Statistical analysis was performed using a one-way analysis of variance followed by Tukey's multiple comparisons test. (G) Schematic of the experimental design and analytical approaches. (H) Single-cell transcriptional landscape visualized by t-SNE projection, colored by annotated cell types (left) and experimental condition (right). (I) Feature plots depicting representative marker expression across clusters. (J) Dot plot showing normalized gene expression levels of marker genes associated with common cell types. (K) Sankey diagram showing the cell type composition of different treatment groups.

Berberine administration demonstrated significant efficacy in mitigating DSS-induced colitis, as evidenced by lower body weight loss (Figure 1A-B), reduced disease activity index (DAI) scores (Figure 1C), longer colon length (Figure 1D), and superior intestinal barrier function (Figure 1E) relative to both DSS-induced and positive control groups. Histopathological evaluation through hematoxylin-eosin (HE) staining revealed intact colonic architecture and an absence of significant immune cell infiltration in berberine-treated mice compared to the DSS group (Figure 1F).

To explore the contribution of various cell types in berberine-mediated inflammation therapy, we subjected colonic tissues from colitis mice to single-cell RNA sequencing (Figure 1G). We retained 12,119 cells from 2 colitis samples and 14,930 cells from 3 berberine-treated colitis samples after quality control (Figure S1A). Unsupervised clustering combined with canonical marker-based annotation revealed seven major cell types: epithelial cells, enterocytes, T cells, B cells, myeloid cells, stromal cells, and plasma B cells (Figure 1H-J, S1B). We observed marked changes in cell population structures between groups (Figure 1K, S1C). Epithelial cells and enterocytes constituted an average of 77% of all cells recovered in the berberine group but only 46% in the DSS group. Immune cells accounted for 19% in the berberine group, while in the DSS group, they reached 42%. These observations prove that the DSS group exhibited higher infiltration levels of immune cells in colon tissue than the berberine group.

**Figure 2:**
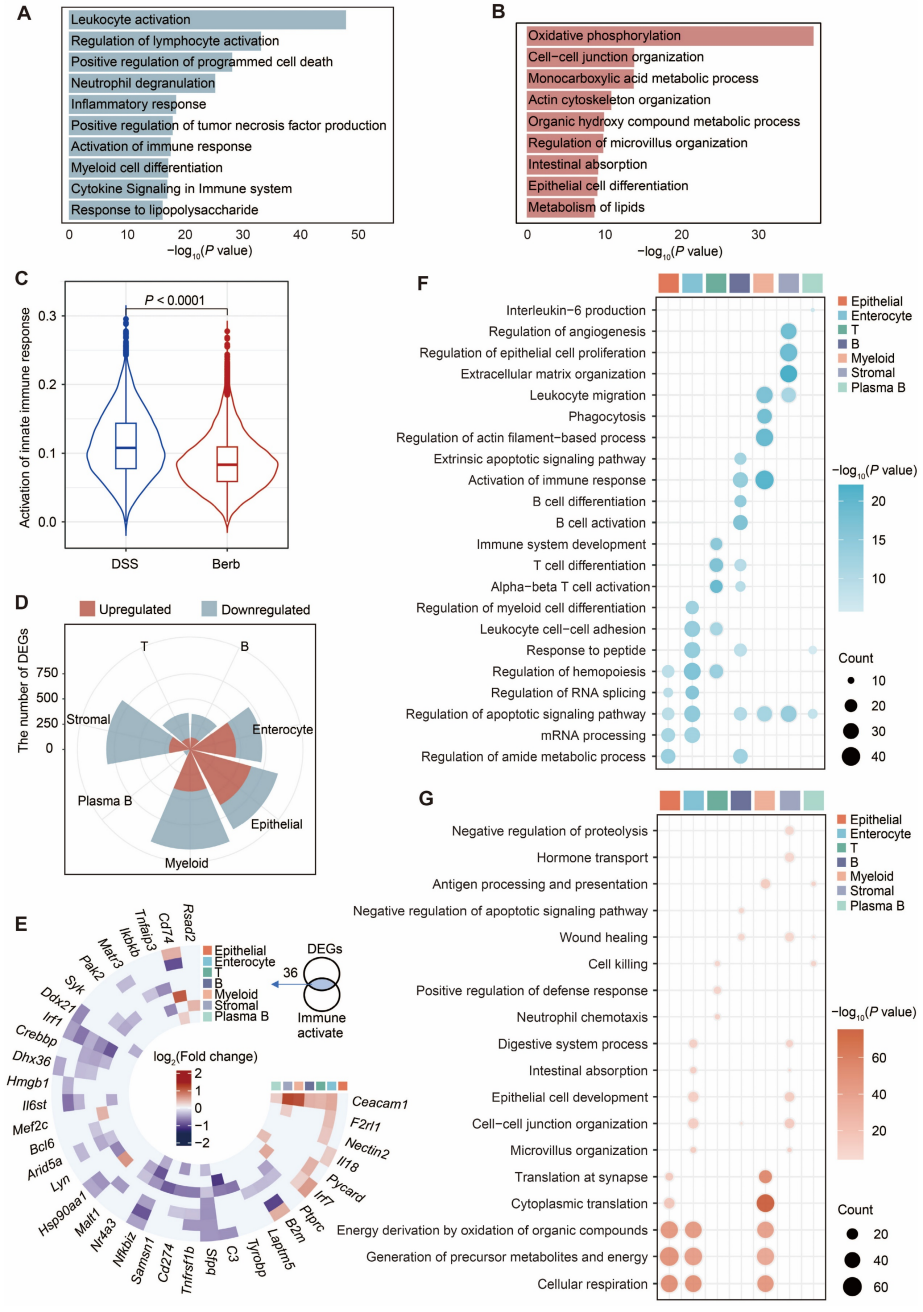
Aberrant gene expression profiles in berberine group compared with DSS group. Bar plots displaying the enrichment of upregulated (A) and downregulated (B) pathways in the berberine group. (C) Violin plot showing the scores of the activation innate immune response pathway across groups. (D) Rose diagram illustrating the number of differentially expressed genes (DEGs) by cell type. (E) Intersection matrix mapping shared regulatory targets between cell type-specific DEGs and innate immune activation pathways. Dot plot showing the representative gene ontology enriched in upregulated (F) or downregulated (G) genes in each cell type. All differential comparisons mentioned refer to the berberine/DSS group. *P* value < 0.05 was defined as statistically significant.

To investigate the potential mechanism of berberine in DSS-induced colitis, we performed enrichment analysis of the differentially expressed genes (DEGs) in Berb/DSS group. Upregulated genes showed predominant enrichment in oxidative phosphorylation, intercellular junction organization, organic metabolic processes, and intestinal absorption pathways (Figure 2A). The genes downregulated in berberine were primarily enriched in leukocyte activation and innate immune response pathways (Figure 2B). Consistent with this finding, AUC scoring of the innate immune response pathway revealed significantly higher activity in the DSS group compared to the berberine group (Figure 2C).

Cell population-specific expression patterns revealed both shared and unique regulatory targets. Integration of cell type specific DEGs (Figure 2D) with immune activation pathway genes identified shared regulatory targets (Figure 2E). Core immune modulators including *Arid5a*, *Il6st*, *Nfkbiz*, and *Sfpq* displayed marked downregulation across multiple cell types, while a few genes were upregulated, including *Ceacam1* (known to activate CD8^+^ T cells 30). Functional mapping demonstrated epithelial-lineage populations (epithelial cells, enterocytes, stromal cells) in berberine-treated group exhibited enhanced tight junction formation and mitochondrial energy metabolism (Figure 2F). In contrast, immune cells showed attenuated functional profiles, with suppressed pathways governing T cell activation/differentiation and leukocyte migration (Figure 2G). Based on these colitis-linked functional alterations, we conducted in-depth mechanistic studies on key cell types.

### Berberine improves epithelial metabolic competence and colonic integrity

Epithelial cells and enterocytes constituted the predominant populations in colonic specimens. Due to the inherent fragility of enterocytes during tissue dissociation, these cells exhibited lower normalized count RNA and feature RNA values in our single-cell dataset (Figure S1D), which prompted focused analysis on epithelial cells.

**Figure 3:**
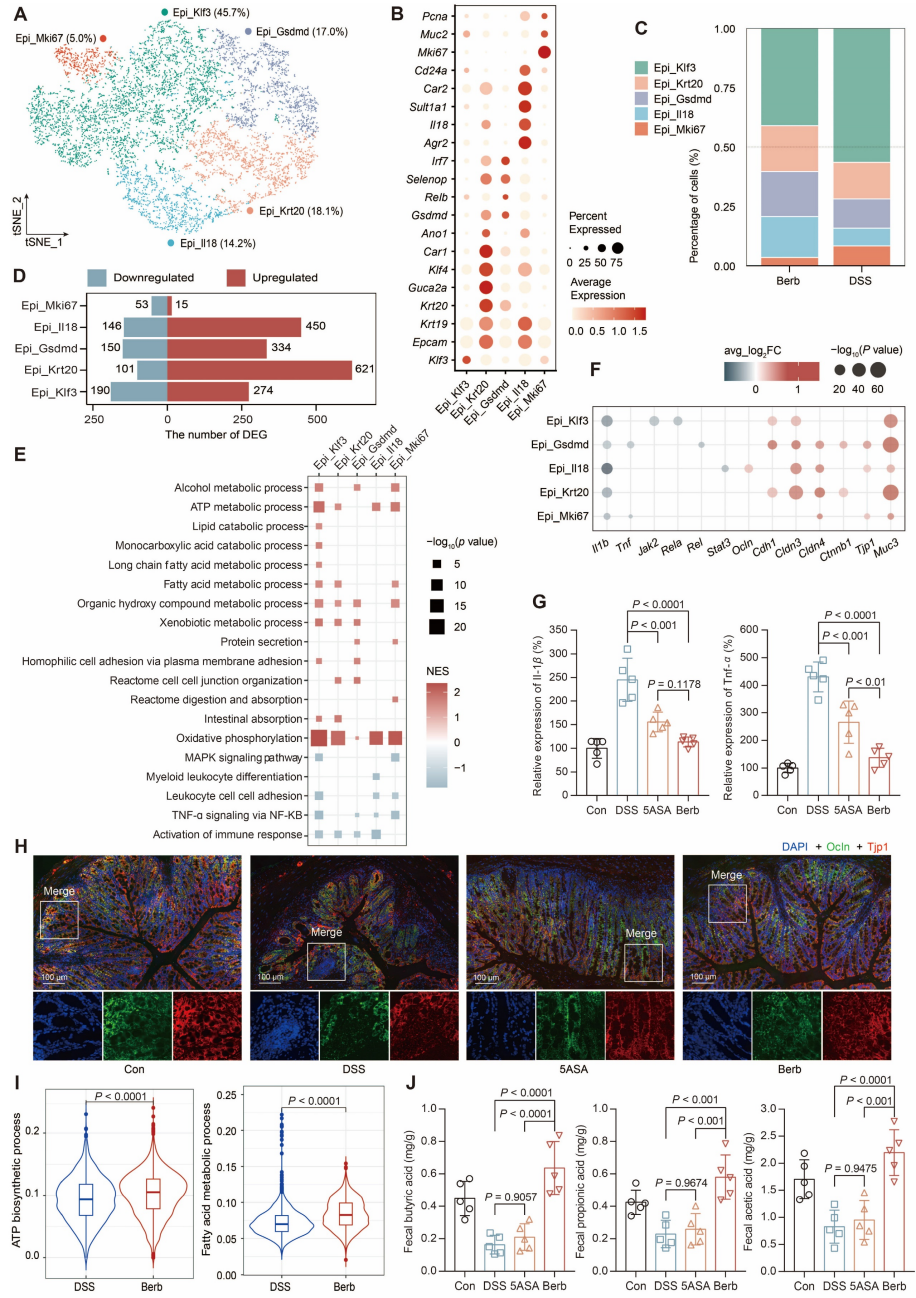
Characterization of epithelial subtypes and associated gene expression profiles. (A) t-SNE visualization of five epithelial subtypes. (B) Dot plot displaying gene expression levels of representative genes for each subtype. (C) Cellular composition of subtypes in the berberine and DSS groups. (D) The number of differentially expressed genes by subtype. (E) GSEA analysis of epithelial cell function-related pathways. NES: normalized enrichment score. (F) Differential expression of tight junction and immune activation pathway-related genes across subtypes. (G) Concentrations of Il-1*β* and Tnf-*α* cytokines in mouse colon tissues. (H) Immunofluorescence showing the expression of cadherin 1 and ZO-1 in colon tissues. (I) Violin plot illustrating pathway scores across groups. (J) Concentrations of short-chain fatty acids in mouse feces. *P* value < 0.05 was defined as statistically significant.

Subsequent separate sub-clusterings on epithelial cells resulted in five fine-grained cell subsets, which were all characterized by their distinctive expression profile and annotated based on their marker gene (Figure 3A-B, S2A-B). The four subtypes, which accounted for the majority (Figure 3C), exhibited a high number of upregulated DEGs (Figure 3D). Gene set enrichment analysis (GSEA) of the gene ontology pathways demonstrated that TNF-*α* signaling via NFKB and the immune response of leukocytes were significantly downregulated in the berberine group (Figure 3E). At the gene level, significant downregulation of *Tnf* and *Il1b* was also observed (Figure 3F-G). However, the pathways upregulated in different subtypes were distinct (Figure 3E). For instance, Gsdmd^+^ epithelial cells were enriched in protein secretion and tight junction. Immunofluorescence staining of colonic tissue sections proved that tight junction proteins such as the claudin family 31, occluding (*Ocln*), zonula occludens-1 (ZO-1, *Tjp1*) 32, and cadherin 1 (*Cdh1*) 33 were upregulated in the berberine group (Figure 3H, S2C). Klf3^+^ epithelial cells were enriched in fatty acid metabolism pathways (Figure 3E), while all subtypes showed significant upregulation in oxidative phosphorylation. The AUC scores of the ATP biosynthetic and fatty acid metabolism pathways for the overall epithelial cells also showed that the berberine group scored significantly higher than the DSS group (Figure 3I). One source of energy for epithelial cells is the content of SCFAs in the gut environment. Therefore, we measured the SCFAs content in mouse feces across different groups, showing that berberine supplementation could increase acetic acid, propionic acid, and butyric acid levels in the intestine (Figure 3J).

### Berberine specifically enriches *Akkermansia* in the gut microbiota of DSS-induced colitis mice

**Figure 4:**
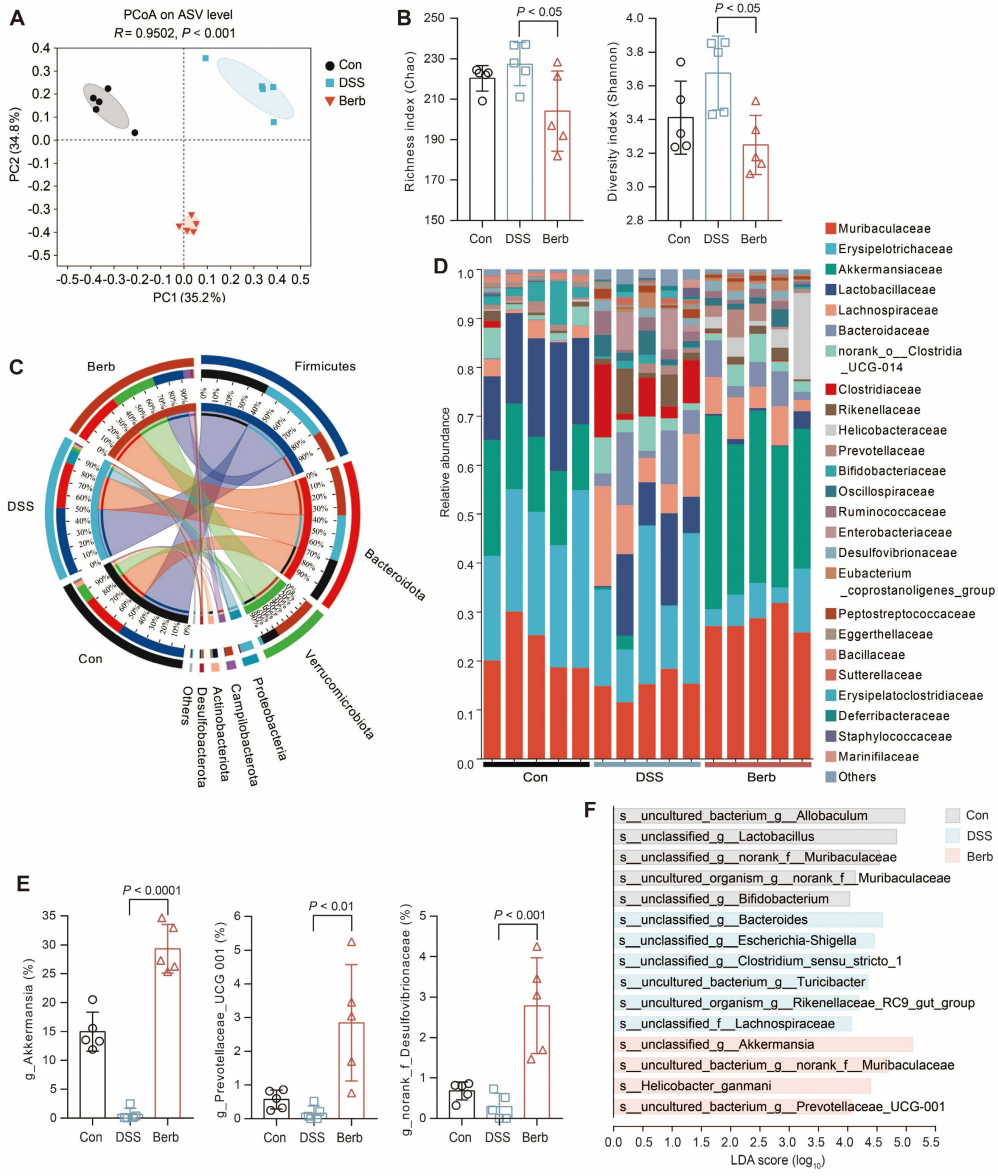
16s rRNA gene sequencing reveals the modulation of gut microbiota by berberine supplementation. (A) Gut microbiome composition on the ASV level was visualized by PCoA for all groups. The 95% confidence intervals for the species composition of each sample group are represented by the ovals. (B) Community α-diversity was evaluated using the richness (Chao) and diversity (Shannon) indices. (C) Relative abundance of gut bacterial phylum among groups. (D) Relative abundance of gut bacterial family among samples. (E) Genera with significantly altered relative abundance of gut microbial taxa after berberine treatment. (F) LDA scores of differentially expressed species between groups. Data are represented as means ± SD (n = 5), and statistical significance was evaluated by one-way analysis of variance followed by Tukey's multiple comparisons test. *P* value < 0.05 was defined as statistically significant.

Short-chain fatty acids, microbial metabolites derived from gut microbiota, serve as a critical energy source for colonic epithelial cells. To investigate the impact of berberine on gut microbiota composition in a murine colitis model, we performed 16S rRNA gene sequencing on fecal samples. Principal coordinates analysis (PCoA) demonstrated a clear separation between the berberine-treated group and the DSS-induced colitis group (Figure 4A), indicating distinct microbial community structures. While alpha diversity indices (Shannon and Chao) revealed no significant differences in overall microbial richness or diversity across groups (Figure 4B), berberine administration induced selective enrichment of specific taxa. The phylum *Verrucomicrobiota* showed marked expansion in the berberine group (Figure 4C), with its constituent family *Akkermansiaceae* (Figure 4D) and genus *Akkermansia* (Figure 4E) being significantly elevated. Linear discriminant analysis effect size (LEfSe) further identified unclassified_g_*Akkermansia* as the predominant species-level biomarker in the berberine group (Figure 4F). This aligns with established evidence that *Akkermansia* ameliorates UC through mucin degradation and subsequent SCFAs production, which supports host energy metabolism 34,35. Immunofluorescence staining also indicated that the mucin content in colonic tissue of the berberine group was more intact compared to the DSS group (Figure S2D), which aids in the colonization of *Akkermansia* in the intestine. Berberine treatment suppressed the pathogenic *Escherichia Shigella* complex while enriching unclassified_f_*Desulfovibrionaceae* (Figure 4E). Meanwhile,* Desulfovibrionaceae* is a family known for high acetate production 36. These findings propose that berberine alleviates colitis by selectively modulating gut microbiota to enhance SCFAs generation, thereby improving colonic energy metabolism and maintaining mucosal homeostasis.

Furthermore, experiments involving antibiotic mediated gut microbiota depletion demonstrated that berberine still ameliorated colitis related symptoms even in the absence of microbiome (Figure S3A-D). This indicates that while berberine modulates the gut microbiota, its therapeutic effects are not solely dependent on microbial regulation, underscoring its multi-target pharmacological nature.

### Berberine inhibits the expansion of inflammation-associated fibroblast subset

**Figure 5:**
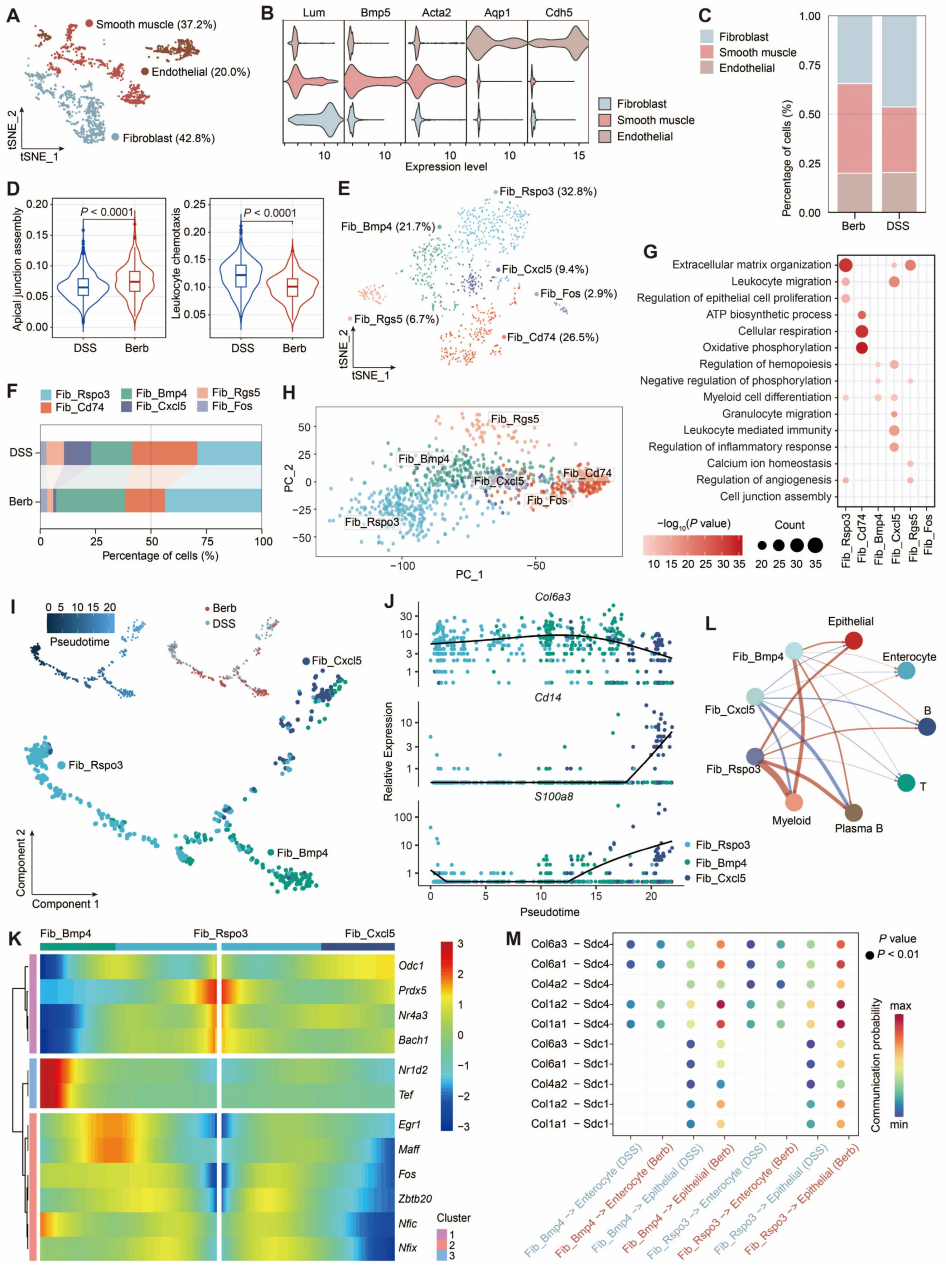
Fibroblast heterogeneity reveals berberine-induced modulation of collagen signaling and immune crosstalk in colitis. (A) t-SNE visualization of stromal subtypes. (B) Gene expression levels of representative markers for fibroblasts, smooth muscle cells, and endothelial cells. (C) Proportional distribution of stromal subtypes across different groups. (D) Violin plot depicting the scores of the apical junction assembly and leukocyte chemotaxis pathways in fibroblasts across groups. (E) t-SNE visualization of six fibroblast subtypes. (F) Sankey diagram illustrating the composition of fibroblast subtypes across different groups. (G) Dot plot representing enrichment of Gene Ontology (GO) terms linked to fibroblast subtypes. (H) Principal component ordination of fibroblast subtype compositional variation. (I) Pseudotime trajectory analysis of three fibroblast subtypes based on group, cell subtypes, and pseudotime. (J) Dynamic expression profiles of key genes along the pseudotime axis. (K) Heatmap showing the expression of transcription factors identified as varying significantly along the pseudotime trajectory. (L) Circle graph illustrating the differential interaction strength between three fibroblast subtypes and other cell types in berberine and DSS groups. Red and blue lines denote upregulated and downregulated interactions in the berberine group (M) Bubble plot displaying increased signaling of collagen-Sdc1/4 pairs between three fibroblast subtypes and other cell types in the berberine group.

Single-cell analysis identified three major stromal populations: fibroblasts (*Lum*), smooth muscle cells (*Acta*2), and endothelial cells (*Cdh5*) (Figure 5A-B). Fibroblast proportions were significantly elevated in the DSS group (Figure 5C). Given the dual roles of fibroblasts in tissue architecture and immune regulation 37,38 (Figure S4A), we assessed their functional activity using AUC scoring. This revealed distinct pathway activation patterns between groups, particularly in apical junction assembly and leukocyte chemotaxis (Figure 5D). Based on these findings, we focused on fibroblasts and performed separate sub-clusterings to obtain six fine-grained cell subsets (Figure 5E, S4B). Among these, Cxcl5^+^ fibroblasts were primarily found in the DSS group (Figure 5F, S4D). GO enrichment analysis of the top 500 most variable genes within Cxcl5^+^ fibroblasts revealed their specific functions are associated with leukocyte-mediated immunity (Figure 5G).

Co-expression of *Il1b* and* Il11* within this subset suggested acquisition of an immune phenotype 39,40 (Figure 5E, S4C). Furthermore, functional overlap was observed between Cxcl5^+^ fibroblasts and other subsets (Rspo3^+^ and Bmp4^+^ fibroblasts). Principal component analysis (PCA) confirmed clear transcriptional divergence among these subsets (Figure 5H).

Pseudotemporal trajectory analysis (Monocle) revealed bifurcating differentiation paths originating from Rspo3⁺ fibroblasts, diverging toward either Bmp4⁺ or Cxcl5⁺ subsets (Figure 5I). These two cellular differentiation pathways expressed distinct transcription factors, with *Maff*, *Fos*, and *Zbtb20* in Bmp4^+^ fibroblasts, and *Odc1* and *Zeb2* in Cxcl5^+^ fibroblasts (Figure 5K). Early pseudotime phases were marked by structural genes (*Col6a3*), while terminal phases expressed immune markers (*Cd14, S100a8*) (Figure 5J). Moreover, DSS group exhibited intensified Cxcl5⁺ fibroblast-immune cell crosstalk, predominantly mediated by CD44 signaling (Figure 5L, S4E). In contrast, berberine promoted fibroblast-epithelial interactions via collagen-Sdc1/4 ligand-receptor pairs (Figure 5M), suggesting a mechanistic shift toward mucosal repair.

### Berberine reduces *Il1β* expression to inhibit innate immunity in myeloid cells

**Figure 6:**
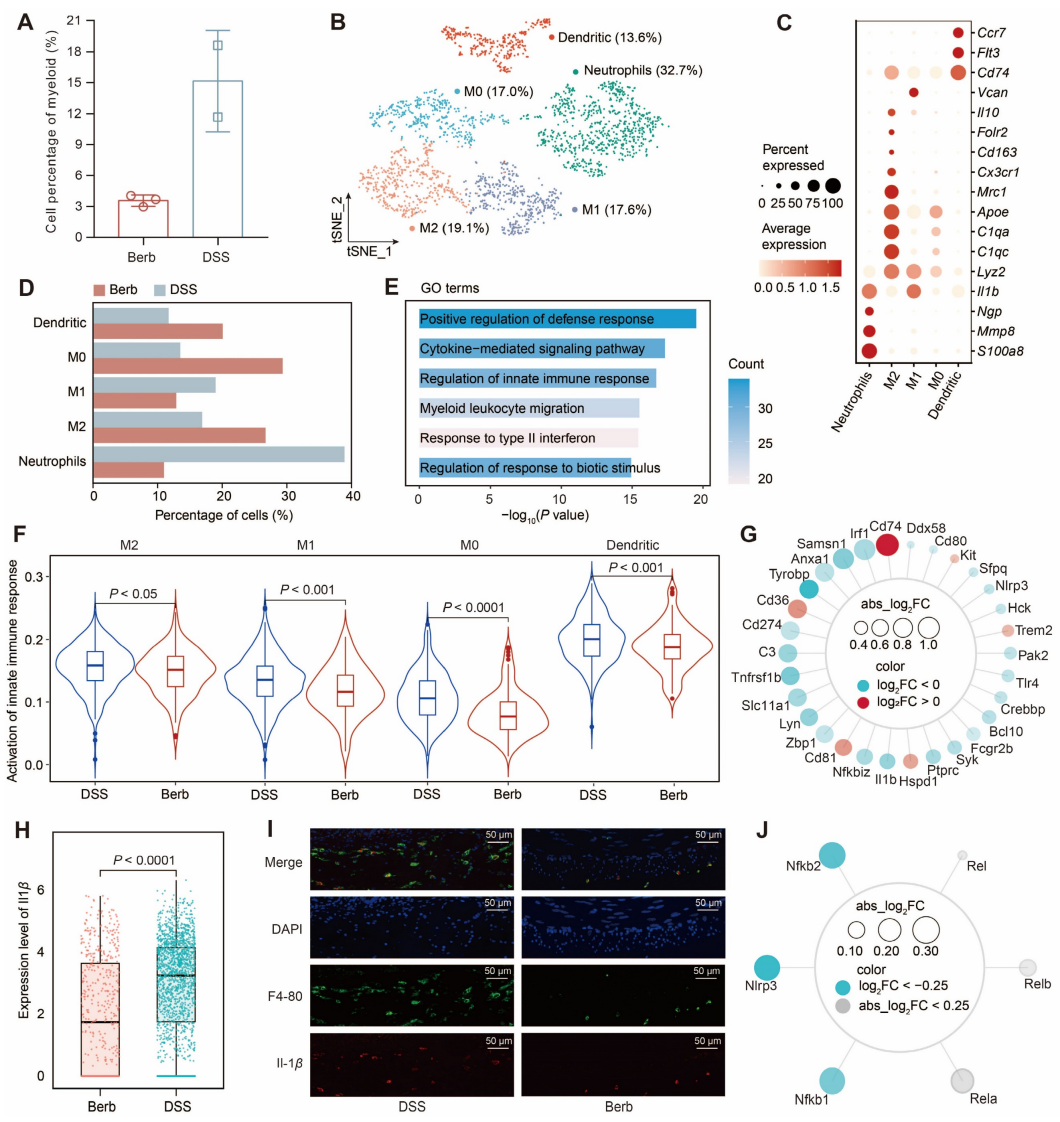
Myeloid heterogeneity reveals *Il1β* driven innate immune dysregulation and its modulation by berberine in colitis. (A) Comparative abundance of myeloid cells in berberine and DSS groups. (B) t-SNE visualization of myeloid cell heterogeneity, resolving five distinct subtypes. (C) Dot plot displaying the gene expression levels of representative genes for each subtype. (D) Cellular composition of subtypes in the berberine and DSS groups. (E) Gene Ontology (GO) enrichment of downregulated genes in neutrophils from the berberine group. (F) Violin plot showing the activation of the innate immune response pathway score across different myeloid subtypes. (G) Radar chart mapping overlaps between differentially expressed genes (DEGs) and innate immune pathway components in myeloid cells. (H) *Il1β* expression levels in myeloid subtypes. (I) Immunofluorescence imaging of macrophage infiltration and Il-1*β* expression in colon tissues. (J) Radar chart profiling upstream regulators of *Il1β* expression in myeloid cells.

The proportion of myeloid cells in the berberine group was significantly lower than that in the DSS group (Figure 6A). Subsequent separate sub-clusterings on myeloid cells (Figure 6B), guided by canonical marker genes (Figure 6C) and literature-curated gene signatures 41 (Figure S5A), obtained five distinct subsets: neutrophils, M2 macrophages, M1 macrophages, M0 macrophages, and dendritic cells. The ratio of neutrophils and M1 macrophages in the berberine group was significantly lower than in the DSS group, while the proportions of M0 and M2 macrophages were higher in the berberine group (Figure 6D).

Neutrophils dominated the myeloid compartment in DSS group. GO enrichment analysis of the DEGs between neutrophils in the berberine and DSS groups indicated an upregulation of apoptosis signaling regulation and a downregulation of innate immune response and response to stimuli (Figure 6E, S5B-C). Consistently, AUC scoring confirmed heightened innate immune pathway activity across myeloid subtypes in DSS mice (Figure 6F). We intersected the DEGs from myeloid cells with genes related to the innate immune response pathway to obtain shared genes (Figure 6G). A few genes were upregulated in the berberine group, including *Cd74, Cd36, Cd81, Hspd1, Trem2,* and *Kit,* with *Cd74* being associated with inhibiting macrophage migration. Among the downregulated genes were the pro-inflammatory factor *Il1b* and the tumor necrosis factor receptor *Tnfrsf1b*. The expression of *Il1b* in the myeloid cells of the DSS group was significantly higher than that in the berberine group (Figure 6H). Using F4-80 to locate macrophages in the mouse colon (Figure 6I), we also observed a high expression of Il-1b in the DSS group. Further investigation into the upstream gene expression of *Il1b* revealed that berberine inhibits the expression of *Il1b* by suppressing the inflammasome (*Nlrp3*) and NF-κB signaling pathways (*Nfkb1* and *Nfkb2*) in myeloid cells (Figure 6J, S5D). This finding was further validated in cultured murine macrophage Raw 264.7 cells, where berberine treatment significantly reduced the LPS-induced upregulation of *Il1b* and *Nlrp3* at the mRNA level (Figure S6). This coordinated inhibition of upstream signaling cascades underpins berberine's anti-inflammatory effects in myeloid cells.

### Berberine intervention on T cells and myeloid cells interaction

**Figure 7:**
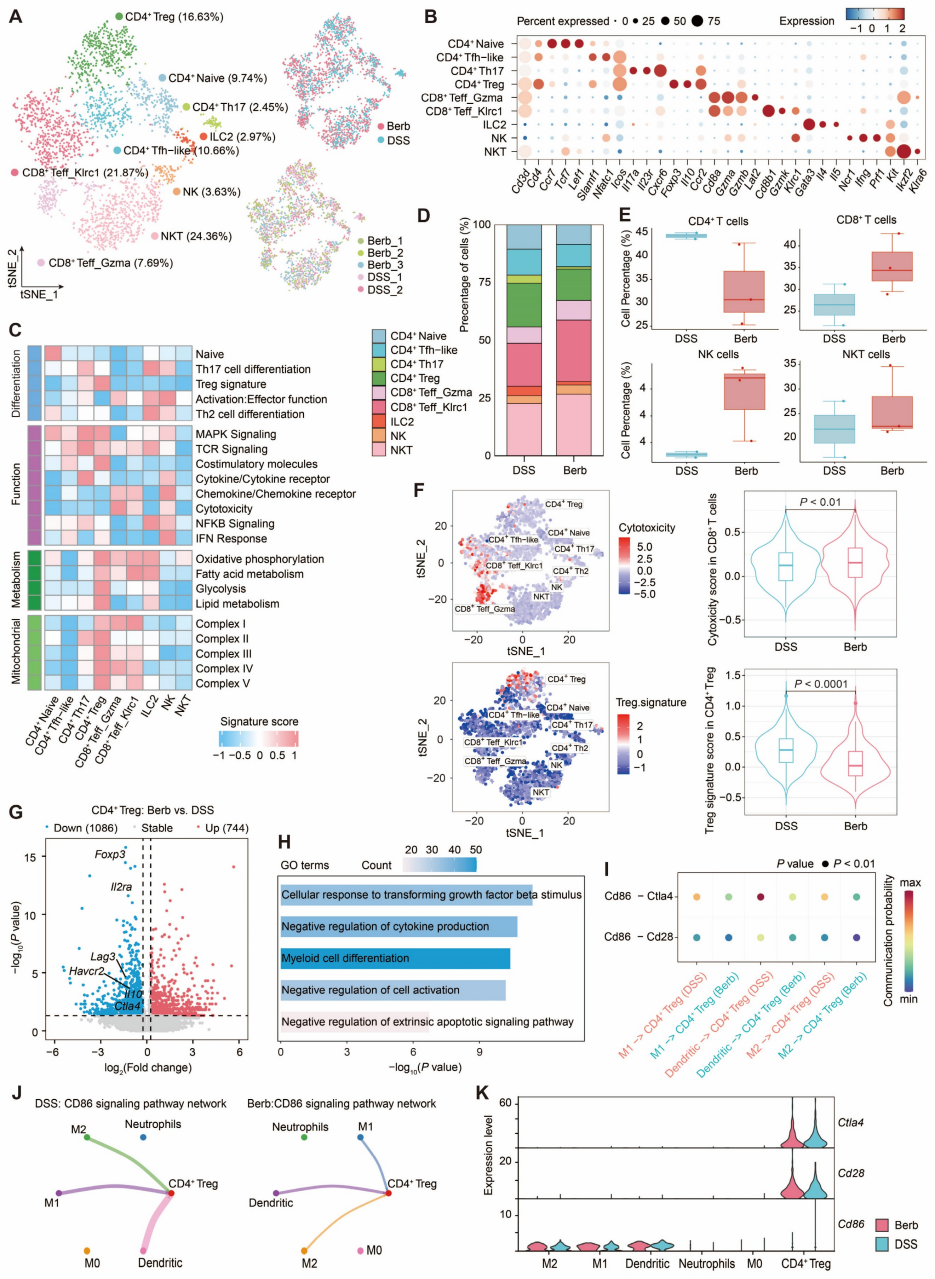
Single-cell transcriptomic profiling of T cell subtypes reveals functional dynamics and myeloid crosstalk in colitis. (A) t-SNE visualization of T cell subtypes with their annotation (right) and experimental conditions (left). (B) Expression levels of marker genes defining each T cell subtype. (C) Proportional distribution of T cell subtypes across different groups. (D) Comparative abundance of CD4^+^ T cells, CD8^+^ T cells, NK cells, and NKT cells among groups. (E) Functional enrichment scores associated with T cell subtypes. (F) Cytotoxicity and Treg signature enrichment: intra-subtype heterogeneity (left) and inter-group comparisons (right) (G) Volcano plot depicting differentially expressed genes in CD4^+^ Treg between berberine-treated and DSS-treated groups. (H) Gene Ontology (GO) term enrichment analysis of downregulated genes in CD4^+^ Treg cells. (I) Bubble plot displaying decreased signaling of CD86-Cd28/Ctla4 interaction pairs between CD4^+^ Treg cells and myeloid subtypes in the berberine group. (J) Circle graph illustrating the CD86 signaling pathway network between CD4^+^ Treg cells and myeloid subtypes in berberine and DSS groups. (K) Expression profiles of *CD86*, *Cd28*, and *Ctla4* for CD4^+^ Treg cells and myeloid subtypes.

We investigated T cell populations captured in a colonic tissue scRNA-seq dataset to gain deeper insights into their cellular phenotypes and the mechanisms underlying their roles in immune regulation and inflammation. Bioinformatic analysis of gene expression and functional enrichment through reclustering revealed nine major subclusters: natural killer T cells (NKT), natural killer cells (NK), group-2 innate lymphoid cells (ILC2), CD8^+^ Klrc1^+^ T effector cells (Teff), CD8^+^ Gzma^+^ Teff, CD4^+^ regulatory T cells (Treg), CD4^+^ T helper type 17 cells (Th17), CD4^+^ T follicular helper-like cells (Tfh-like), and CD4^+^ naïve cells (Figure 7A-C).

CD4^+^ Treg demonstrated pronounced enrichment of energy metabolism-associated gene signatures (Figure 7C), a finding corroborated by elevated mitochondrial complex gene signature scores indicating heightened activation in the colitis model. Comparative analysis revealed greater CD4^+^ Treg abundance in the DSS group relative to berberine-treated counterparts (Figure 7D). While CD4^+^ T cells predominated in DSS group, berberine intervention correlated with increased proportions of CD8^+^ T cells and NK T cells (Figure 7E), populations recognized for cytotoxic activity against infected or malignant cells. This cytotoxic phenotype was particularly pronounced in CD8^+^ T cells from berberine-treated samples (Figure 7F). In contrast, Treg signature, such as *Foxp3*, *Havcr2*, *Il10,* and *Ctla4*, showed low expression levels in the berberine group (Figure 7G). GO analysis of downregulated genes revealed CD4^+^ Treg related to myeloid cell differentiation (Figure 7H), suggesting that berberine intervention may alter the communication between CD4^+^ Treg and myeloid cells. CellChat-based intercellular interaction analysis between CD4^+^ Treg and myeloid cells demonstrated the strongest interaction potential in the CD86 signaling pathway (Figure 7I, S7A-B), particularly in the DSS group. Within this pathway, CD4^+^ Treg function as target cells regulated by M1 macrophages, M2 macrophages, and dendritic cells (Figure 7J, S7C-F). From the perspective of gene expression, CD4^+^ Treg cells express *Cd28* and *Ctla4*, while M1 macrophages, M2 macrophages, and dendritic cells express *Cd86* (Figure 7K).

### Potential drug targets for berberine in counteracting immune cell activation

**Figure 8:**
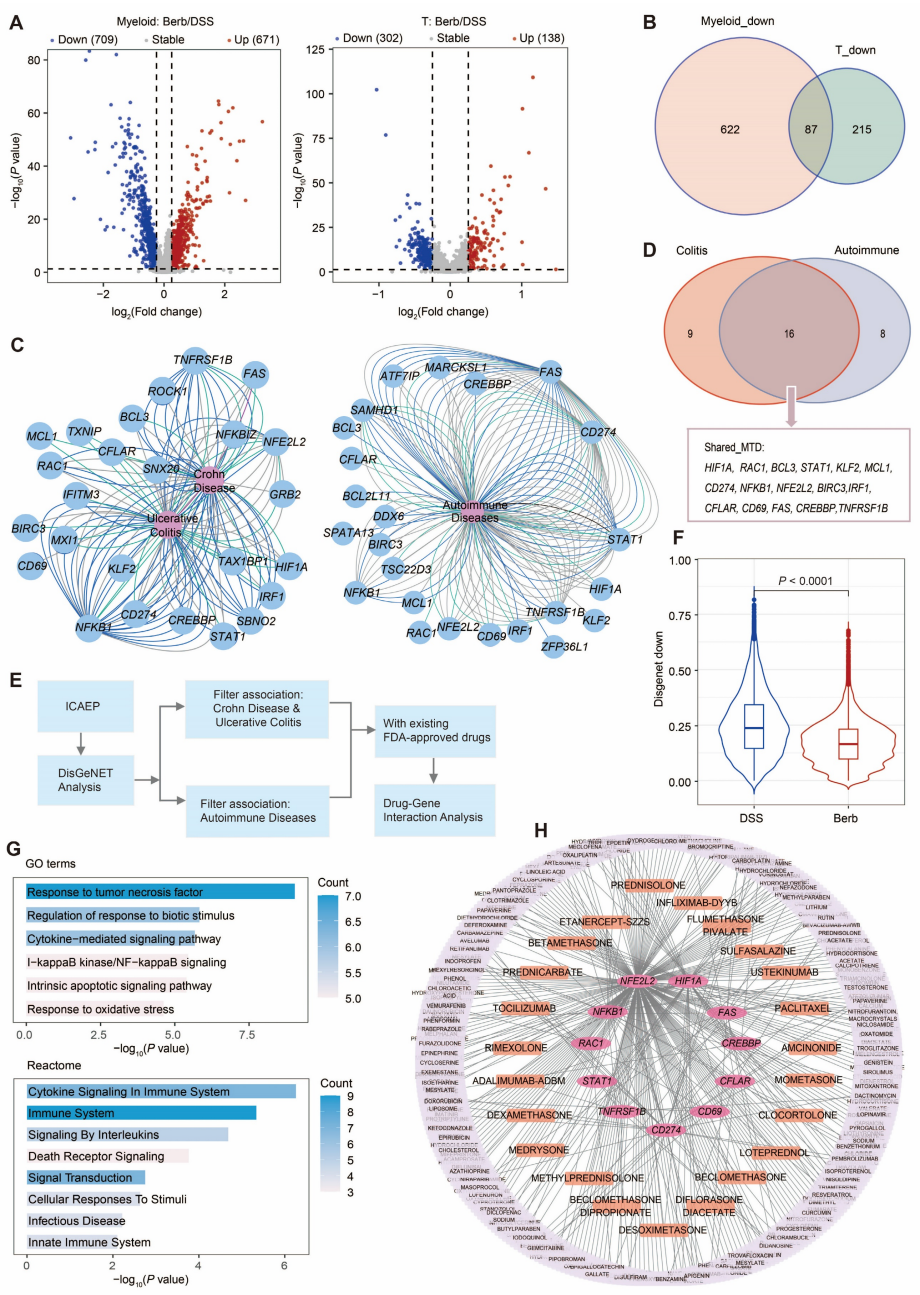
Association of gene regulation and drug interactions of berberine on immune cells. (A) Volcano plot showing the result of differential gene expression analysis between berberine and DSS group in myeloid and T cells. (B) Venn diagram illustrating the 87 downregulated gene signature shared by myeloid and T cells. This signature is termed ICAEP. (C) Network visualizations depict disease-gene interaction maps for ICAEP genes related to inflammatory bowel disease (left) and autoimmune diseases (right). Genes are blue; diseases are pink. (D) Venn diagram illustrating the 16 key genes derived from ICAEP (Shared_MTD) that are involved in both colitis and autoimmune disease. (E) Overview of the workflow for drug-gene interaction analysis. (F) Violin plot illustrating the Shared_MTD signature scores across groups. (G) Pathway enrichment analysis of Shared_MTD was conducted using GOBP and Reactome, respectively. (H) Drug-gene interaction network visualization of all the druggable targets in Shared_MTD. The pink nodes are gene targets, whereas the others are FDA-approved drugs. The orange nodes denote the drugs utilized for anti-inflammatory purposes.

To characterize the molecular mechanisms underlying immune cell hyperactivation and the suppressive effects of berberine on inflammatory responses, we examined dysregulated genes in myeloid and T cells. We identified a shared immune cell activation expression program (ICAEP) based on downregulated differentially expressed genes in Berb/DSS common to both myeloid and T cells. We identified 709 downregulated genes in myeloid cells and 302 in T cells (Figure 8A), with 87 genes constituting the ICAEP (Figure 8B). The ICAEP signature was highly activated in the DSS group (Figure S8A).

REACTOME and Gene Ontology Biological Process (GOBP) pathway analyses of ICAEP signature revealed significant enrichment in innate immunity pathways, including lipopolysaccharide response, tumor necrosis factor signaling, and cytokine-mediated signaling pathways (Figure S8B). To evaluate clinical relevance, DisGeNET analysis established disease-gene interaction networks linking ICAEP components to UC and autoimmune disorders (Figure 8C). Of the 87 genes comprising the ICAEP, 25 were associated with colitis, 24 were mapped to autoimmune diseases, and 16 (Shared_MTD) were associated with both (Figure 8D).

We conducted a target and drug screening analysis within Shared_MTD (Figure 8E). The expression level of Shared_MTD in the DSS group was significantly higher than that in the berberine group (Figure 8F). To determine how the Shared_MTD can contribute to the development of colitis, we performed a pathway enrichment analysis. Consistent with our initial analysis, cytokine signaling in the immune system, response to tumor necrosis factor, and response to biotic stimulus were enriched by this signature (Figure 8G). Of the 16 genes assessed, 11 have at least one FDA-approved drug associated with them (Figure 8H). A total of 362 FDA-approved drugs were identified as potentially interacting with these 11 targets, with 23 of these drugs indicated for anti-inflammatory purposes (Table S2). According to the drug gene interaction database (DGIdb), these 11 genes can be classified into the following 12 categories: clinically actionable, druggable genome, enzyme, transcription factor, transporter, drug resistance, kinase, transcription factor complex, external side of plasma membrane, tyrosine kinase, cell surface, and protease (Figure S8C).

## Discussion

The pathogenesis of UC involves multiple contributing factors, including genetic predisposition 42, dietary influences, gut microbial dysbiosis, and immune hyperactivation 43. Utilizing single-cell sequencing technology, we systematically evaluated cellular composition, gene expression profiles, and intercellular communication from berberine-treated diseased mice. Our analysis further revealed that the therapeutic effect of berberine extends beyond direct modulation of colonic cells, encompassing regulation of gut microbiota composition and enhancement of SCFAs levels in the intestinal environment. This multi-target efficacy highlights the potential of berberine as a comprehensive therapeutic agent for treating the multifactorial pathogenesis of UC. This study confirmed the remarkable efficacy of berberine in alleviating colitis symptoms through oral administration in DSS-induced colitis mice.

Analysis of single-cell data from epithelial cells revealed that the energy metabolism of the berberine group was significantly higher than that of the DSS group. This metabolic restoration carries particular significance given the established relationship between colonic energy deprivation and pathogenic sequelae: insufficient ATP production drives epithelial cell death and tight junction destabilization 44, compromising barrier integrity to initiate cycles of bacterial invasion and inflammation. Supporting this mechanistic framework, fecal SCFA quantification showed marked elevation in berberine-treated cohorts (Figure 3J), providing a biochemical basis for the observed metabolic enhancement 45,46. This result suggests that berberine likely exerts regulatory effects on SCFA-producing gut microbiota. Consistent with this hypothesis, 16S rRNA gene sequencing revealed that berberine specifically enriched the abundance of *Akkermansiaceae* (SCFAs-producer) 47,48 and *Desulfovibrionaceae* (acetic acid-producer) 36 within the gut microbial community.

Stromal cells play a crucial role in maintaining colonic structural integrity. Among them, fibroblasts exhibiting dual functionality regulate the extracellular matrix by providing scaffolding and biochemical support for cells and tissues. They are also influenced by the surrounding microenvironment, differentiating into immune regulators during inflammation 49. Through sub-clustering of fibroblasts, we identified a lymphoid-like fibroblast subset, which we named Cxcl5^+^ fibroblasts. This cell population constituted 12% of the fibroblasts in the DSS group, whereas it accounted for only 1% in the berberine group (Figure S4D), suggesting that berberine suppresses the expansion of Cxcl5^+^ fibroblasts. The gene expression profile of Cxcl5^+^ fibroblasts showed co-expressed pro-inflammatory cytokines of *Il1β* and *Il11*, while GO enrichment analysis highlighted their predominant association with immune-related pathways (Figure 5G). Pseudotime trajectory analysis suggested that Cxcl5^+^ fibroblasts may originate from Rspo3^+^ fibroblasts. With the intervention of berberine, Rspo3^+^ fibroblasts tend to differentiate into Bmp4^+^ fibroblasts. Furthermore, Bmp4^+^ fibroblasts in the berberine group exhibited enhanced collagen-Sdc1/4 signaling, a pathway critical for modulating epithelial barrier integrity and promoting mucosal regeneration in experimental colitis (Figure 5M) 50,51.

Berberine exhibited a potent inhibitory effect on Il-1*β* production, affecting the previously mentioned Cxcl5^+^ fibroblasts and immune cells. Il-1*β*, a key pro-inflammatory cytokine implicated in autoimmune disorders and various cellular processes 52, is primarily produced by innate immune cells and can promote the differentiation of Th17 cells 53. We observed lower levels of Th17 cells in the berberine group among the T cell subpopulations, compared to the DSS group (Figure 7D). Transcriptional profiling revealed that berberine downregulated *Il1β* expression in myeloid cells by inhibiting the inflammasome (*Nlrp3*) and NF-κB signaling pathways (*Nfkb1*, *Nfkb2*) (Figure 6J). This suppression was corroborated by reduced NF-κB pathway activity scores (Figure S5E). This innate immune modulation manifested as decreased neutrophil and M1 macrophage infiltration without compensatory CD4^+^ Treg expansion (Figure 7D), positioning berberine as a selective suppressor of innate-driven inflammation.

Berberine, a multi-target natural therapeutic agent, exhibits a favorable safety profile characterized by low systemic toxicity but poor oral bioavailability 54,55. Despite this pharmacokinetic limitation, its broad mechanism of action confers distinct advantages compared to conventional immunosuppressants. Unlike highly specific biological agents such as anti TNF-*α* monoclonal antibodies (e.g., infliximab 56, adalimumab 57), which act on a single pathway and are associated with risks of immunogenic reactions and secondary loss of response, berberine exerts synergistic effects across multiple inflammatory pathways and cell types, thereby potentially reducing the likelihood of complete non response. Furthermore, whereas biological therapies are characterized by high costs and require parenteral administration, berberine represents an orally administered and cheap alternative. Our drug-target interaction analysis identified 23 approved drugs, including natural product derived chemotherapeutic agents such as paclitaxel 58, monoclonal antibodies (e.g., infliximab-DYYB, adalimumab-ADBM), and synthetic anti-inflammatory drugs (e.g., amcinonide, dexamethasone), which share molecular targets with berberine (Figure 8H). This network pharmacology perspective not only reinforces the multi-target nature of berberine but also implies its potential utility as an adjunctive therapy to improve efficacy or reduce dosage requirements of conventional biologics, as well as a promising alternative treatment option in resource-limited settings.

In summary, this study systematically delineates the multi-target therapeutic mechanisms of berberine in colitis intervention through integrated modulation of immune-stromal-microbial networks. Berberine restores intestinal homeostasis by enriching specific gut microbiota (e.g., *Akkermansia*), thereby enhancing colonic epithelial energy metabolism and barrier integrity. Concurrently, it suppresses innate immune hyperactivation via attenuation of myeloid cell infiltration, inhibition of IL-1*β* production, and blockade of inflammation-associated fibroblast subset differentiation and expansion. The identification of overlapping molecular targets with existing immunotherapies highlights berberine's potential as either a cost-effective monotherapy or a synergistic agent for combination strategies in managing multifactorial inflammatory diseases.

## Supplementary Material

Supplementary methods, figures and table.
