# Supplementary material for: Berberine suppresses colon inflammation via integrated modulation of host metabolism, microbial ecology, and innate immune signaling
Source: Theranostics. 2026 Jan 1;16(4):2019–36. doi: 10.7150/thno.116546 (PMC12680593; doi:10.7150/thno.116546)
Supplement: Supplementary file 1 — Supplementary methods, figures and table. [file thnov16p2019s1.pdf]

**Supplementary Information**

**Berberine suppresses colon inflammation via integrated modulation of host metabolism, microbial ecology, and innate immune signaling**

Yaqin Xiao et al.

Supplementary Methods 2
Supplementary Figure 1 4
Supplementary Figure 2 5
Supplementary Figure 3 6
Supplementary Figure 4 7
Supplementary Figure 5 8
Supplementary Figure 6 9
Supplementary Figure 7 10
Supplementary Figure 8 12
Supplementary Table 1 13

## Supplementary Methods

### Antibiotic treatment

For *in vivo* antibiotic treatment, mice were treated with a broad-spectrum oral antibiotics cocktail including 1 g/L ampicillin (Goldbio, USA), 1 g/L metronidazole (Sigma, USA), 1 g/L neomycin (Sigma, USA), and 0.5 g/L vancomycin (Sigma, USA) in sterile distilled water for 5 days. On the 5th day, after discontinuing the combined antibiotics, the DSS (n = 5) and Berb group (n = 5) were given 2.5% DSS in drinking water for 7 days and were respectively treated daily with water, 50 mg/kg berberine by oral gavage. The Con group (n = 5) received normal water and was administered 0.2 mL of water by oral gavage daily. Mice were sacrificed on the $14^{th}$ day.

### Quantitative real-time PCR

Murine macrophage cell line RAW 264.7 was obtained from American Type Culture Collection (USA). Cells were maintained in complete DMEM supplemented with 10% fetal bovine serum, and 1% penicillin/streptomycin (Cat. #15140122; Thermo Scientific) at 37 °C in a humidified 5% $CO_2$ atmosphere. Cells were plated at a density of $1\times10^5$ cells per well in 6-well plates, and allowed to attach for 12 h. For stimulation, cells were pre-treated with or without 20 μM berberine for 4 hours, followed by the addition of lipopolysaccharides (LPS) to a final concentration of 1 μg/ml for an additional 6 hours before cell collection.

Total RNA was extracted from harvested RAW 264.7 cells using the TaKaRa MiniBEST Universal RNA Extraction Kit (Cat. #9767, TaKaRa), followed by RT-qPCR analysis with the HiScript II One Step qRT-PCR SYBR Green Kit (Cat. #Q221-01, Vazyme). Detailed procedures were performed in accordance with the respective manufacturer's protocols. The PCR primer sequences used for detection are listed below.

Gapdh: 5′-AGAAGGTGGTGAAGCAGGCATC-3′

5′-CGAAGGTGGAAGAGTGGGAGTTG-3′

Nlrp3: 5′-AGCCTTCCAGGATCCTCTTC-3′

5′-CTTGGGCAGCAGTTTCTTTC-3′

Il-1β: 5′-CTGCTTCCAAACCTTTGACC-3′

5′-AGCTTCTCCACAGCCACAAT-3′

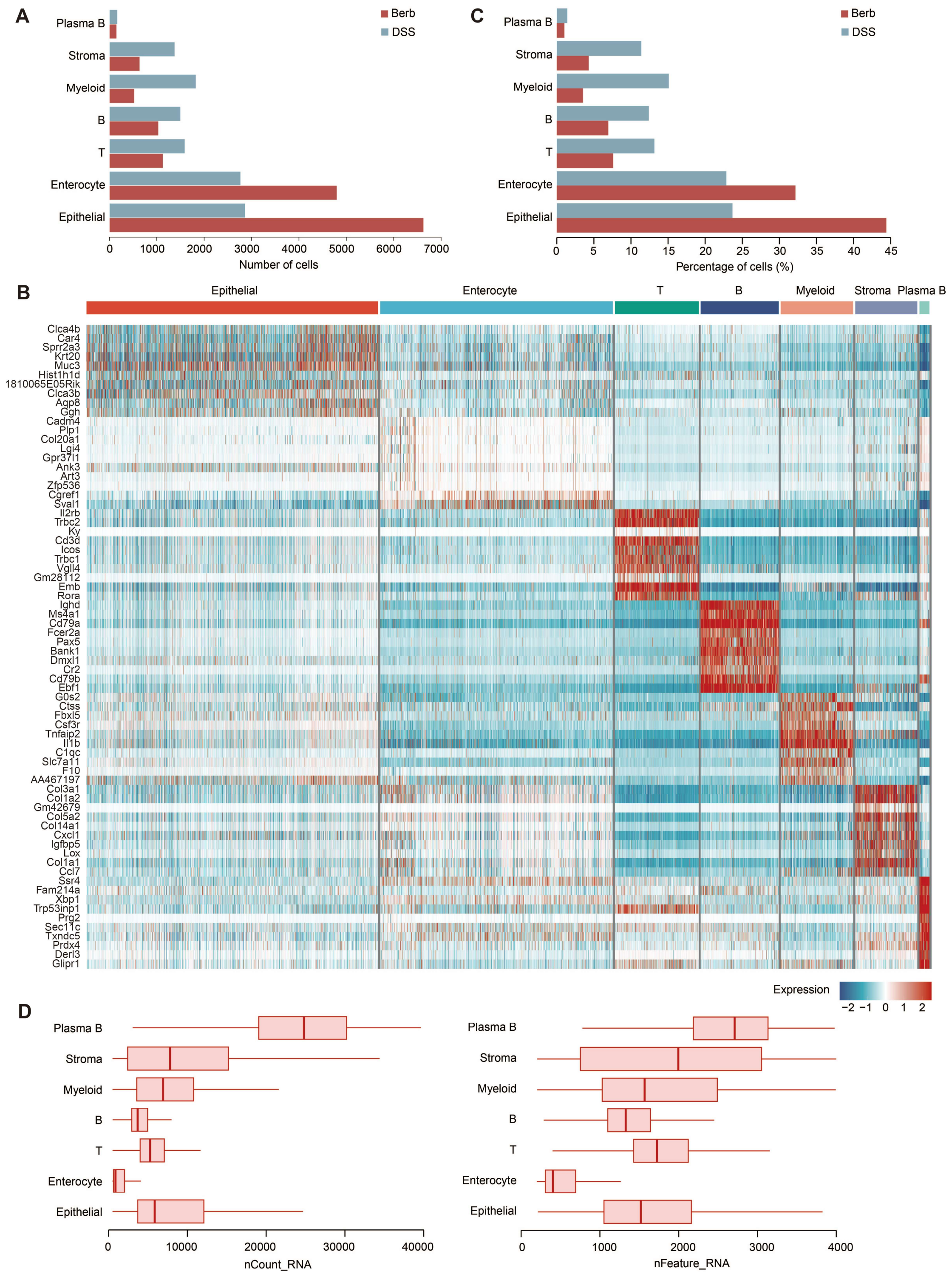


**Supplementary Figure 1. Data comparison of cell types in berberine and DSS groups.** (A) Number of each cell type in the berberine and DSS groups. (B) Heatmap showing normalized gene expression levels of the top 10 genes associated with each cell type. (C) Composition of each cell type in the berberine and DSS groups. (D) Normalized count RNA and feature RNA characterization in each cell type.

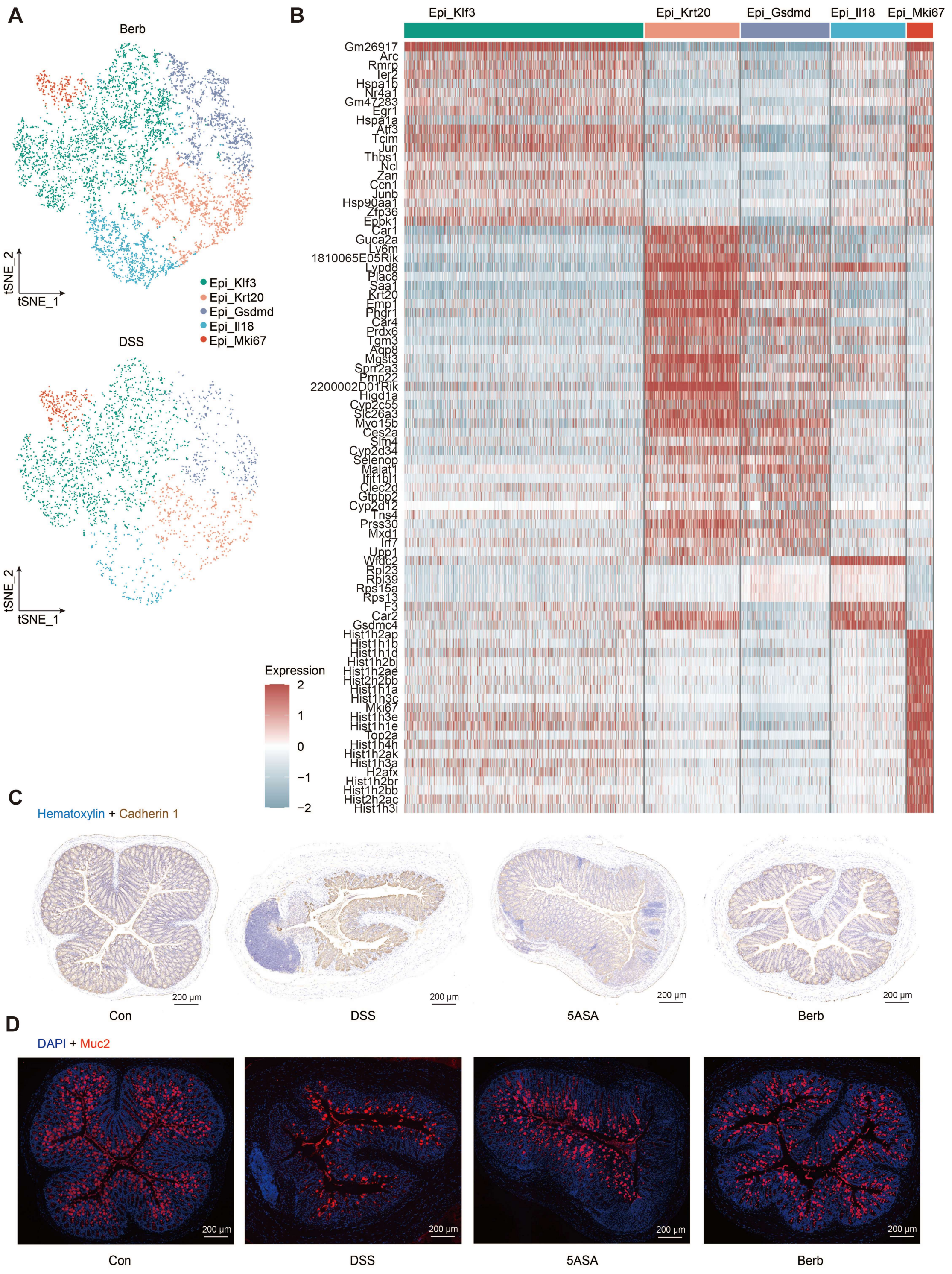


**Supplementary Figure 2. Gene expression profile of epithelial cells.** (A) t-SNE visualization of epithelial cell subtypes under different experimental conditions. (B)Heatmap showing normalized gene expression levels of the top 20 genes associated with epithelial cell subtypes. Immunohistochemistry and immunofluorescence revealing the expression of Cdh1 (C) and Muc2 (D) in colon tissues.

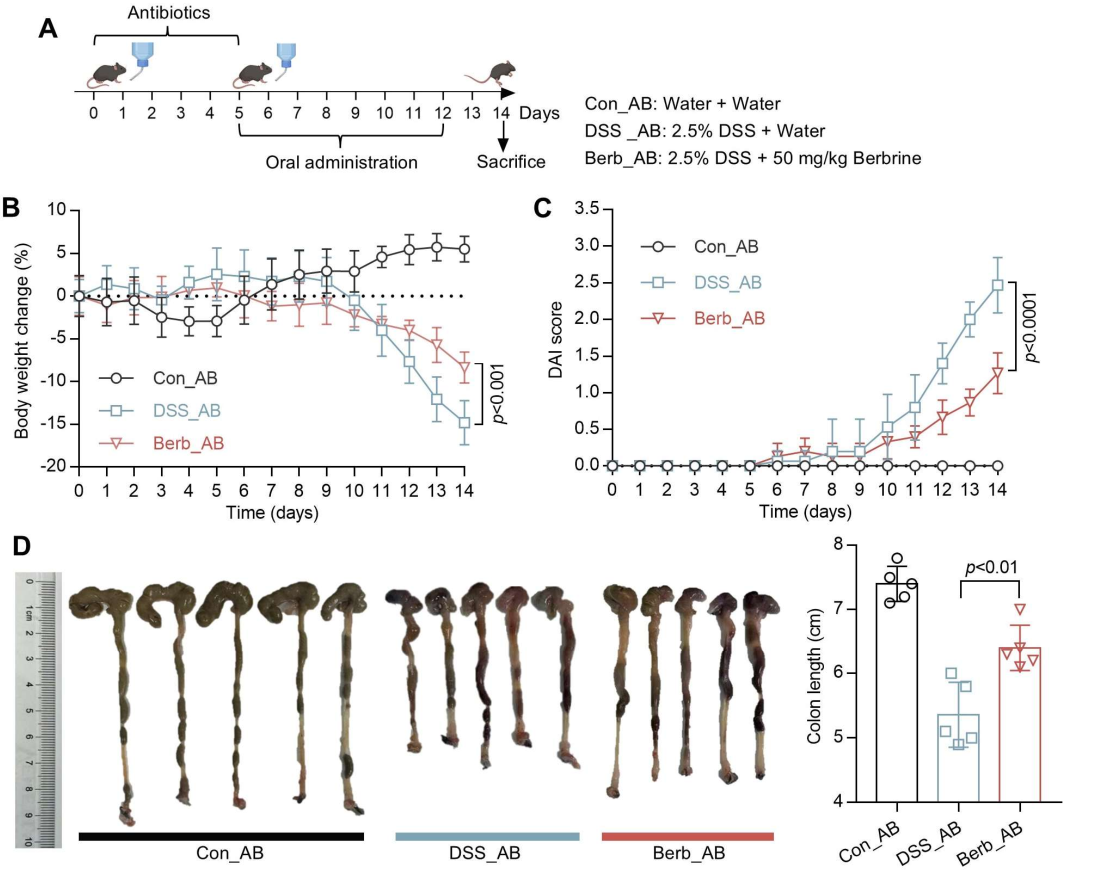


**Supplementary Figure 3. Antibiotic mediated microbiota depletion experiment.** (A) C57BL/6J mice were pretreated for 5 days with broad-spectrum oral antibiotics added to the drinking water and then provided with water, or 2.5% DSS-containing water for 7 days, or with FOS and levan treatments as described in the methods section (n = 5). Body weight (B) and DAI score (C) were measured daily. (D) Colon length was measured after euthanizing the mice. The data are shown as the means ± SD (n = 5). Each experiment was repeated twice. Statistical analysis was performed using one-way analysis of variance followed by Tukey's multiple comparisons test.

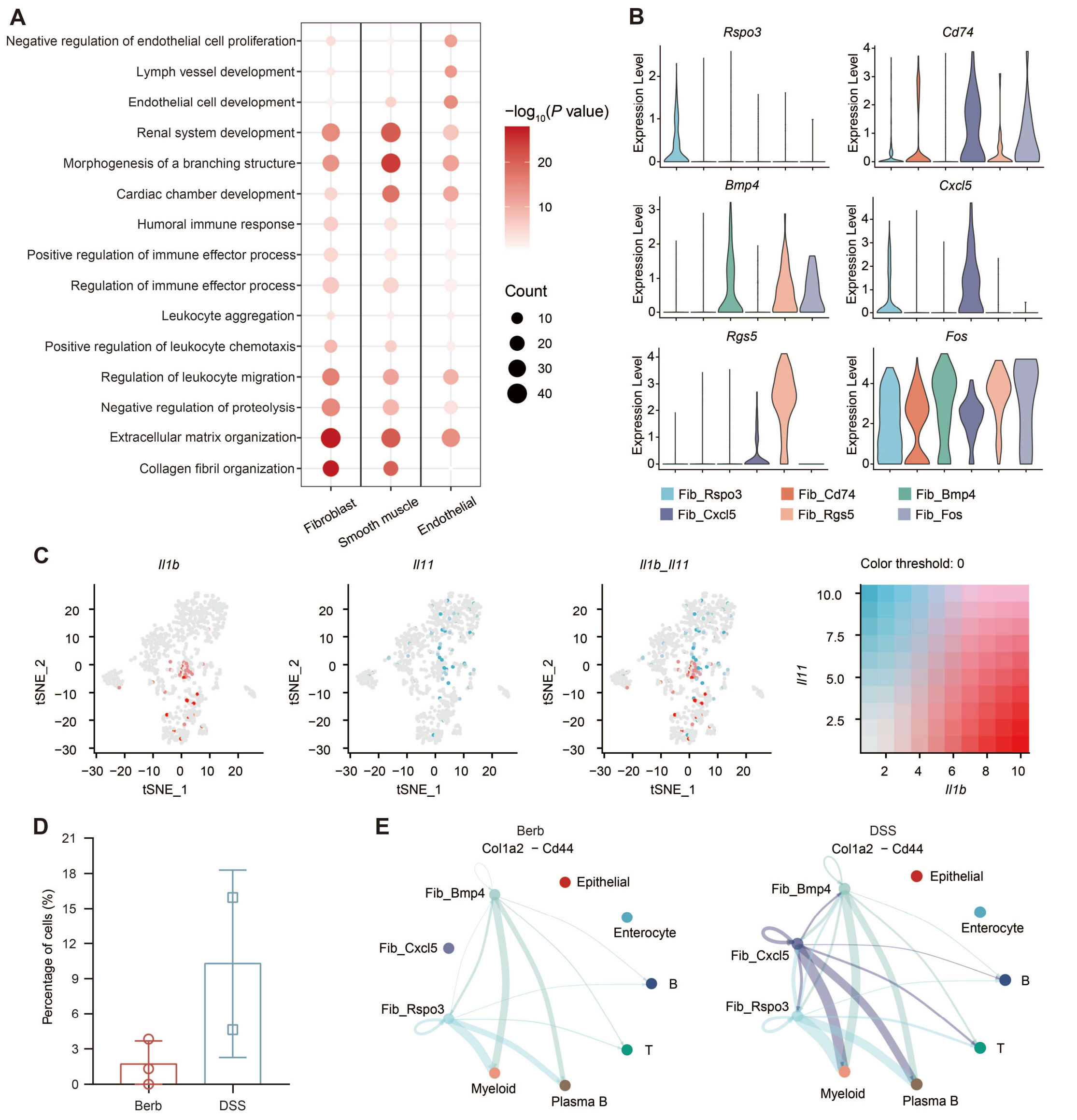


**Supplementary Figure 4. Analysis of fibroblast subtypes.** (A) Dot plot representing the enrichment of gene ontology terms linked to fibroblasts, smooth muscle, and endothelial cells. (B) Gene expression levels of representative markers for fibroblast subtypes. (C) t-SNE visualization showing the co-expression of *Il1β* and *Il11* genes in fibroblasts. (D) Proportion of Cxcl5$^{+}$ fibroblasts among fibroblasts in samples from the berberine and DSS groups. (E) Circle graph illustrating the Col1a2-Cd44 signaling pathway network in berberine and DSS groups.

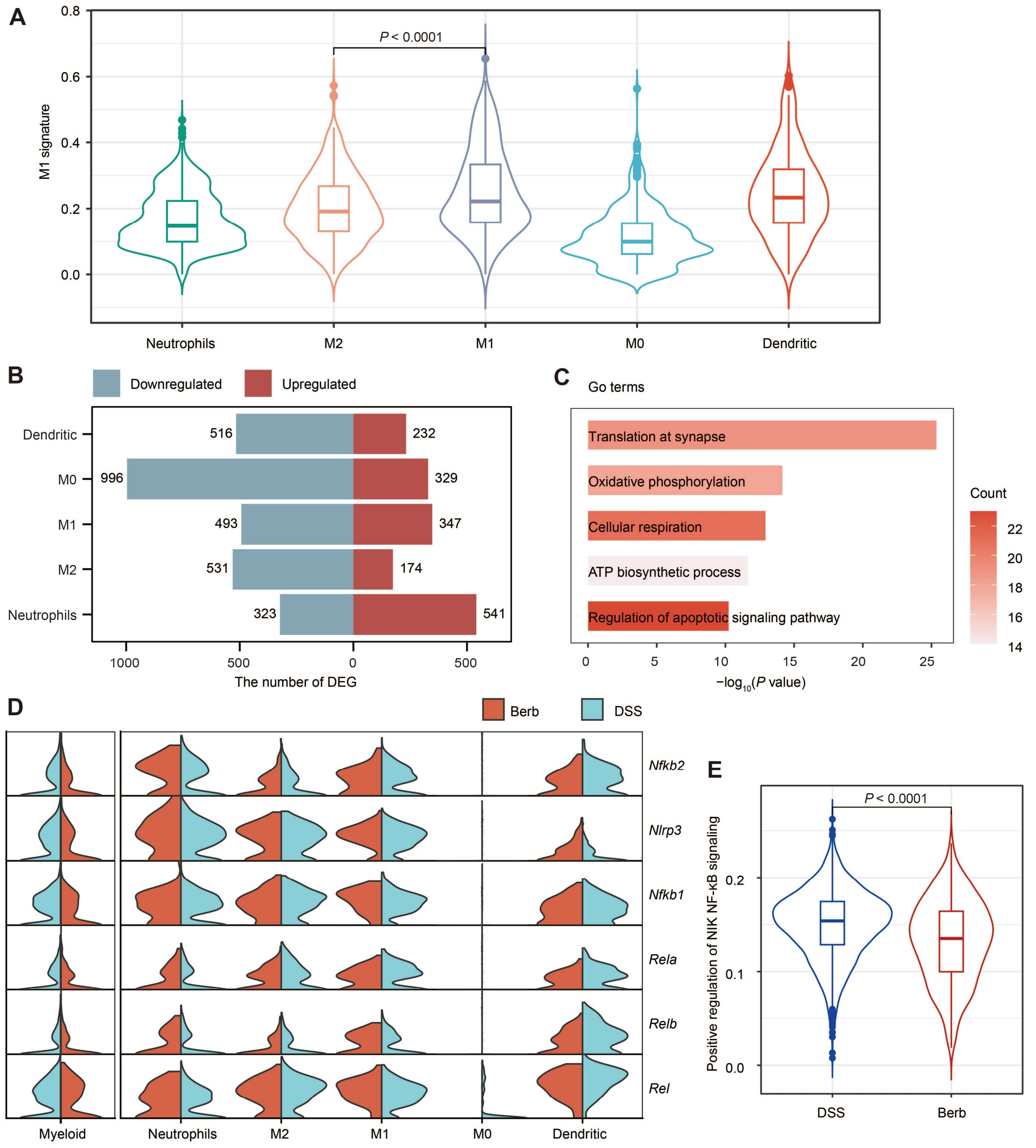


**Supplementary Figure 5. Analysis of myeloid cell subtypes.** (A) M1 signature enrichment scores in myeloid subtypes. (B) The number of differentially expressed genes across myeloid subtypes. (C) Gene ontology enrichment of upregulated genes in neutrophils from the berberine group. (D) Expression profiles of genes for myeloid cells and subtypes in groups. (E) Violin plot showing the scores of positive regulation of the NF-κB signaling pathway among myeloid cells across different groups.

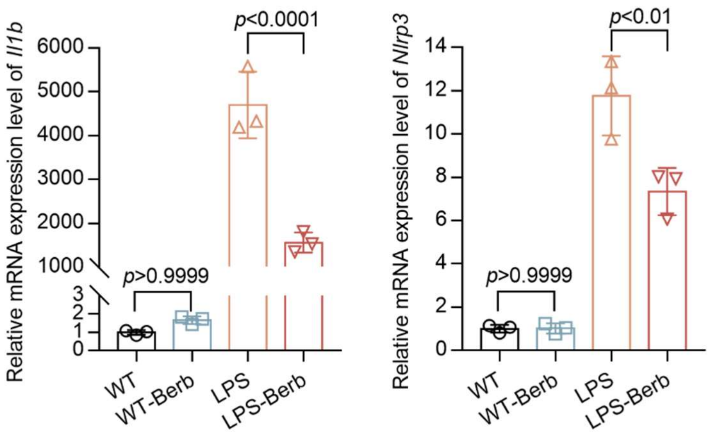


**Supplementary Figure 6. Effect of berberine on *Il1b* and *Nlrp3* mRNA expression in activated RAW 264.7 macrophages.**

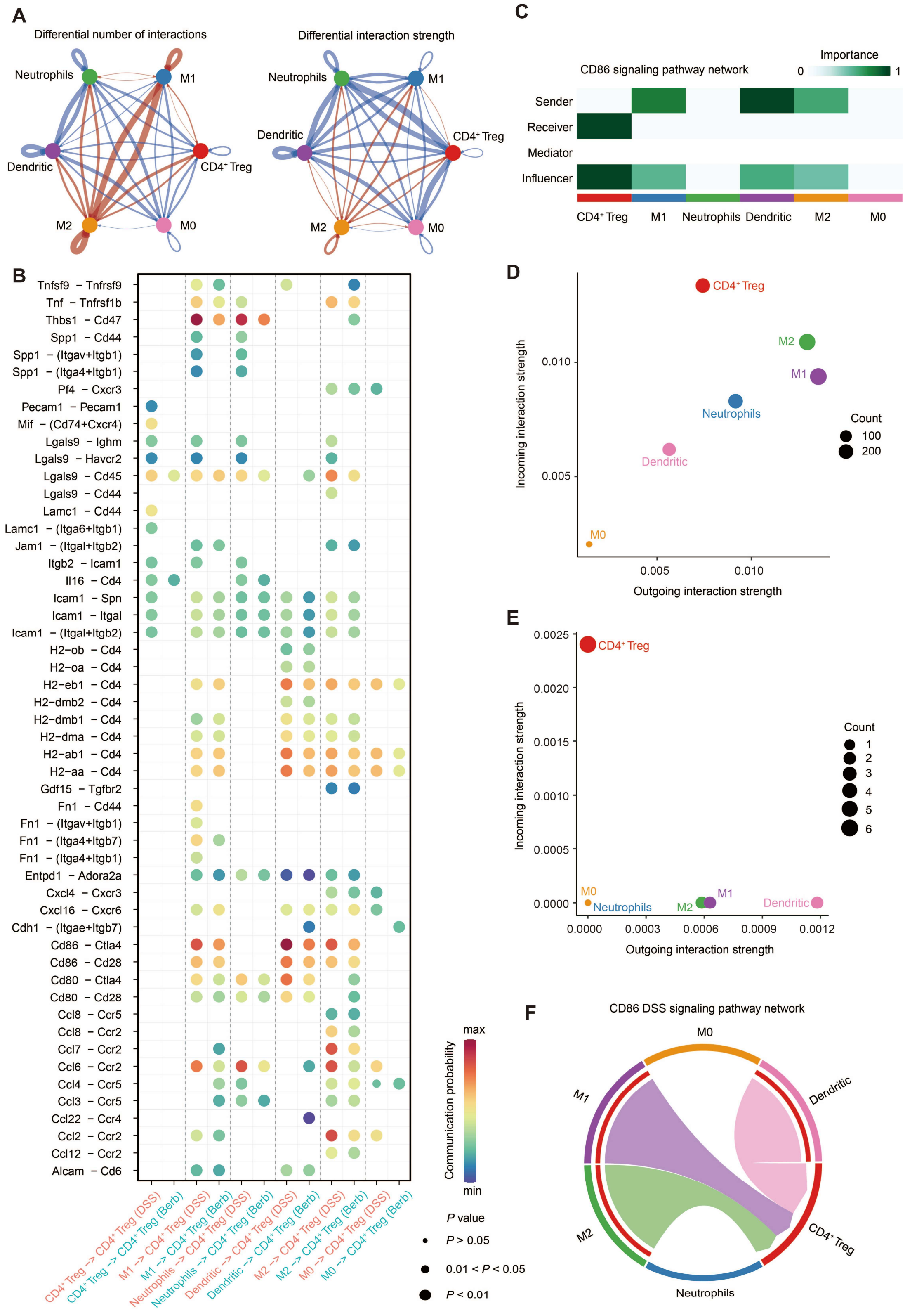


**Supplementary Figure 7. Analysis of CD4$^+$ Treg interactions with myeloid subtypes.** (A) Circle graph illustrating the differential number of interactions and interaction strengths between myeloid subtypes and CD4$^+$ Treg in berberine and DSS

groups. Red and blue lines denote upregulated and downregulated interactions, respectively, in the berberine group. (B) Bubble plot displaying decreased signaling between myeloid subtypes and $CD4^+$ Treg in berberine group. (C) Network centrality scores of CD86 signaling pathway. (D) Cell-cell interaction strengths plots indicating incoming and outgoing interactions for all significant ligand-receptor pairs (upper section) and CD86 signaling pathway (lower section) in DSS group. (E) Chordal graphs illustrating the CD86 signaling cell-cell interaction patterns between myeloid subtypes and $CD4^+$ Treg in the DSS group.

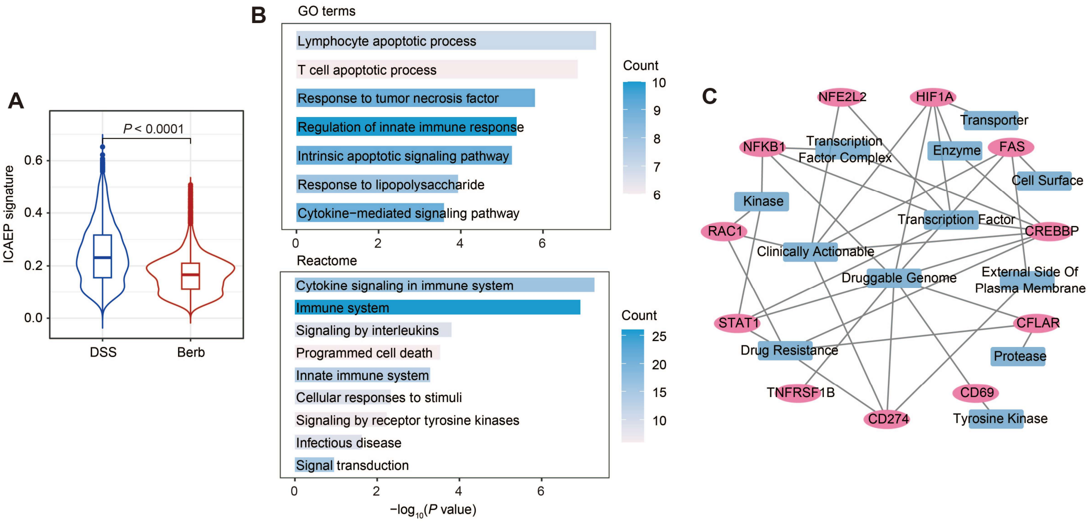


**Supplementary Figure 8. Analysis of ICAEP signature and category-gene interaction network.** (A) ICAEP signature enrichment scores in the DSS and berberine groups. (B) Pathway enrichment analysis of ICAEP conducted using GOBP and Reactome. (C) Category-gene interaction network visualization of 11 genes in Shared_MTD. The pink nodes represent gene targets, whereas the other nodes indicate categories.

**Supplementary Table 1. Number of valid sequences per sample in 16s rRNA sequencing.**

| Sample | ASV_num | Seq_num | Mean±SD of Seq_num |
|---|---|---|---|
| Berb1 | 398 | 109015 | |
| Berb2 | 364 | 98737 | |
| Berb3 | 388 | 126942 | 116654±29154.9 |
| Berb4 | 441 | 161693 | |
| Berb5 | 303 | 86883 | |
| Con1 | 368 | 113023 | |
| Con2 | 416 | 113118 | |
| Con3 | 400 | 112235 | 105497±16498.5 |
| Con4 | 416 | 113118 | |
| Con5 | 355 | 75991 | |
| DSS1 | 457 | 102703 | |
| DSS2 | 322 | 119110 | |
| DSS3 | 411 | 75787 | 114441.2±30166.1 |
| DSS4 | 387 | 115613 | |
| DSS5 | 507 | 158993 | |
